# Comparison of the C-mediating killing activity and C-activating properties of mouse monoclonal and polyclonal antibodies against Trypanosoma cruzi

**DOI:** 10.1155/S0962935192000450

**Published:** 1992

**Authors:** T. L. Kipnis, D. V. Tambourgi, M. J. M. Alves, W. Dias da Silva

**Affiliations:** 1 Departamento de Imunologia ICB Universidade de São Paulo Av. Prof. Lineu Prestes 2415 São Paulo CEP 05508-900 Brazil; 2 Departamento de Bioquimica IQ Universidade de São Paulo Av. Prof. Lineu Prestes 748 São Paulo CEP 00508-970 Brazil; 3 Laboratório de Imunoquimica Instituto Butantan Av. Vital Brazil 1500 São Paulo CEP 05504-900 Brazil

## Abstract

A Mouse polyclonal antiserum against Trypanosoma cruzi or its IgG and IgM fractions and five monoclonal antibodies (two IgM, two IgG_1_ and one IgG_2a_) recognize and combine with membrane components of trypomastigote forms of the parasite as revealed by immunofluorescence. Although all these antibodies sensitize trypomastigotes and prepare them to activate the complement (C) system, as measured by consumption of total C, C4, B and C3, only the polyclonal antiserum or its IgG, IgM and Fabμ fragments were able to induce trypanosome lysis by the alternative C pathway.


Mediators of Inflammation, 1, 309–312 (1992)

### Comparison of the C-mediating killing activity and C-activating properties of mouse monoclonal and polyclonal antibodies against *Trypanosoma cruzi*

**T. L. Kipnis,[1,CA] D. V. Tambourgi,[1] M. J. M. Alves[2] and W. Dias da Silva[3]**

[1] Departamento de Imunologia, ICB, Universidade de São Paulo Av. Prof. Lineu Prestes 2415, São Paulo, CEP 05508-900;
[2] Departamento de Bioquímica, IQ, Universidade de São Paulo Av. Prof. Lineu Prestes 748, São Paulo, CEP 00508-970;
[3] Laboratório de Imunoquímica, Instituto Butantan Av. Vital Brazil 1500, São Paulo, CEP 05504-900, Brazil

[CA] Corresponding Author

**A MOUSE polyclonal antiserum against *Trypanosoma cruzi* or its IgG and IgM fractions and five monoclonal antibodies (two IgM, two $IgG_1$ and one $IgG_{2a}$) recognize and combine with membrane components of trypomastigote forms of the parasite as revealed by immunofluorescence. Although all these antibodies sensitize trypomastigotes and prepare them to activate the complement (C) system, as measured by consumption of total C, C4, B and C3, only the polyclonal antiserum or its IgG, IgM and Fab$\mu$ fragments were able to induce trypanosome lysis by the alternative C pathway.**

**Key words:** Anti-*T. cruzi* lytic antibodies, Complement activation, Complement regulatory proteins, *Trypanosoma cruzi*

### Introduction

Trypomastigote forms of *Trypanosoma cruzi* (TF), a non-replicative and non-activator of alternative complement pathway (ACP), can be experimentally transformed into activators of ACP upon treatment with proteolytic enzymes,[1] heating at 45°C[2] or incubation with some types of specific immunoglobulins.[3] These observations indicate that the forms of trypomastigotes, during their differentiation either from epimastigotes (EF) (the replicative, non-infective activators of ACP found in the gut of the insect vector) or from amastigotes present essentially inside the cells of the vertebrate hosts, synthesize and express on their cell surface regulatory proteins of C activation (RPC) which are biologically similar to those found on vertebrate cells or plasma.[4,5] Activation of the C system depends on the formation of the C3 convertases, C4b2a and C3bBb.[6] These two bimolecular enzyme complexes activate C3 and some of the C3b formed in turn attaches to the target. In plasma factor H,[7] C4-binding protein (C4bp)[8] and on tissue, C receptor type one (CR1),[9] decay-accelerating factor (DAF)[10] and membrane cofactor protein (MCP)[5] restrict the activity of C4b and C3b and thereby inhibit C activation. Among these RPC a DAF-like glycoprotein, cognominated T-DAF, has been described on the *T. cruzi* cell surface[2,11,12] and recently has been partially cloned.[13]

Patients with Chagas' disease, or mice experimentally infected with *T. cruzi*, produce antibodies that can be detected by the conventional immunological methods,[14] some of them, but not all, being able to prepare, *in vitro*, the TF for C-mediating lysis through ACP.[15] The fragments $F(ab')_2$, Fab' and Fab prepared from these immunoglobulins retain that activity even when the ability to serve as acceptors for C3b is restrained by additional reduction and alkylation of the Fd interchain disulphide bonds.[15] Therefore, the C-mediated lysis of TF by immunoglobulins (Igs), thought to be induced through the ACP, is restricted to some antibody populations and depends on the presence of an intact Fc portion and accessible acceptors for C3b on their Fab moieties. Thus, the transformation of TF into ACP activating particles by those Igs could be related to the epitopes recognized rather than to the Igs isotype. In this report selected monoclonal and polyclonal antibodies anti-*T. cruzi* were used to sensitize TF and the ability of sensitized trypomastigotes to activate C and to become susceptible to C attack are illustrated. These results reinforce the idea that the Igs inducing trypomastigote C-mediating lysis block some RPC-like molecules present on the parasite cell surface.

© 1992 Rapid Communications of Oxford Ltd

### Materials and Methods

*Sera, mAbs, IgM, IgG and Fabμ:* Immune mouse sera (IMS) were obtained from mice 12 weeks after an infection with $1 \times 10^3$ of TF and contained Abs anti-*T. cruzi* as assayed by ELISA (1:1200), indirect immunofluorescence (IIF) (1:50), C fixation reaction (+ + +) and by the ability to sensitize TF for C-mediating lysis. Normal human serum (NHS), normal mouse serum (NMS), and normal guinea pig serum (NGpS) were obtained from individuals or animals devoid of specific Abs for *T. cruzi* assayed as above. Monoclonal antibodies A1 (IgM mAb H3G6), A2 (IgM mAb L1D10), A3 (IgG mAb L2B11), A4 (IgG2a mAb 6A2), A5 (IgG1 mAb B10.1)[16] and the polyclonal IgG (A6) and IgM (A8) and its Fabμ (A9) from IMS (A7), were prepared in our laboratories. Goat anti-mouse IgG or IgM conjugated with FITC (Sigma Chemical Corp., USA) and rabbit serum (A) anti-sheep red blood cells ($E^s$) were also prepared in our laboratories.

*Parasite:* TF (Y strain), free of antibodies against *T. cruzi*, were obtained from blood of mice pretreated with cyclophosphamide (200 mg/kg). Five days after an infection with $1.5 \times 10^5$ *T. cruzi*, the animals were bled and the parasites suspended in Minimum Essential Medium (MEM) and the concentration adjusted to $2.6 \times 10^6$ parasites/$ml^2$.

*Sensitization of trypanosomes with Abs, activation of C system and C-mediating lysis:* One hundred microlitre samples of TF were mixed with equal volumes of NMS, mAbs, IMS or with their IgG, IgM or Fabμ fragments and incubated for 30 min at 4°C.[15] After washing, the presence of specific Igs on the parasite cell surface was examined by IIF technique using anti-Igs-FITC as probe. A volume of NHS as C source was added and the mixture was reincubated for 1 h at 37°C and the number of motile parasites determined using a haemocytometer. The parasites were removed by centrifugation and the supernatants were saved for C titrations. Total C was measured by determining the number of $CH_{50}$ remaining in the samples of NHS incubated with pre-opsonized TF using sheep red blood cells ($E^s$) pre-sensitized with rabbit serum anti-$E^s$ ($E^sA$) in pH 7.4 isotonic veronal buffered saline containing 0.1% gelatin, 15 mM $CaCl_2$ and 5 mM $MgCl_2$ ($VBS^{++}$). The components C4 and C3 were measured by determining the number of haemolytic active molecules (Z) remaining in the samples of NHS incubated with pre-opsonized TF using $E^sAC1$ or $E^sAC142$ intermediate cells, respectively, in $VBS^{++}$ containing 2.5% of glucose ($DGVBS^{++}$).[17] Factor B was measured by determining the number of haemolytically active molecules (Z) remaining in the sample of NHS incubated with pre-opsonized TF using rabbit red blood cells ($E^r$) and NHS previously depleted of factor B in $DGVBS^{++}$ containing 1 mM $Mg^{2+}$ and 10 mM EGTA.[18]

### Results

*Presensitization of trypomastigotes with different antibodies against* T. cruzi*:* The monoclonal antibodies A1 (IgM mAb H3G6), A2 (IgM mAb L1D10), A3 (IgG, mAb L2B11), A4 ($IgG_{2a}$ mAb 6A2), A5 ($IgG_1$ mAb B10.1) and the polyclonal antibodies A6 (anti-*T. cruzi* IgG), A7 (anti-*T. cruzi* chronic mouse serum), A8 (anti-*T. cruzi* IgM) and A9 (anti-*T. cruzi* Fabμ) are able to recognize and combine with epitopes present on the TF cell surface membrane as revealed by IIF. The opsonized trypanosomes presented uniform, linear deposits of mouse IgG or IgM on their surfaces (data not shown).

*Activation of C system by trypomastigotes bearing antibodies on their cell surface:* TF pre-opsonized with the anti-*T. cruzi* antibodies present in the samples when incubated with NHS under conditions allowing activation of either the classical and the alternative C pathway induce an extensive reduction in the number of $CH_{50}$ (Fig. 1A), and in the number of haemolytically active molecules of C4 (Fig. 1B), factor B (Fig. 1C) and C3 (Fig. 1D).

*Ability of specific Abs of mediating* T. cruzi *C lysis:* Trypanosome lysis, as evaluated by comparing the number of motile parasites at the beginning and at the end of the incubation period of pre-opsonized TF with NHS, as C source, was only seen when parasites were presensitized with the polyclonal specific antiserum, or with their Igs, IgG or IgM, and to a lesser extent, with their Fabμ (Table 1).

### Discussion

The *T. cruzi* infection in humans induces the production of Abs directed to several parasite epitopes and belonging to different Ig isotypes.[19] The appearance of these Abs coincides with the first signals of the infection, remaining high during the acute phase of the disease.[20] The Abs capable of sensitizing trypomastigotes for C mediated lysis follow a similar pattern but it has been shown that they become undetectable in some patients treated with therapeutic drugs.[14] These antibodies or their $F(ab')_2$ or Fab fragments as noted earlier, are known to transform trypomastigotes into ACP activators,[15] a suggestion that was reinforced by two recent observations: first, the demonstration that trypomastigotes express a cell surface glycoprotein, T-DAF, that inhibits the assembly of C3 convertases;[2,11–13] and secondly, the verification that IgM, the most powerful antibody class to activate

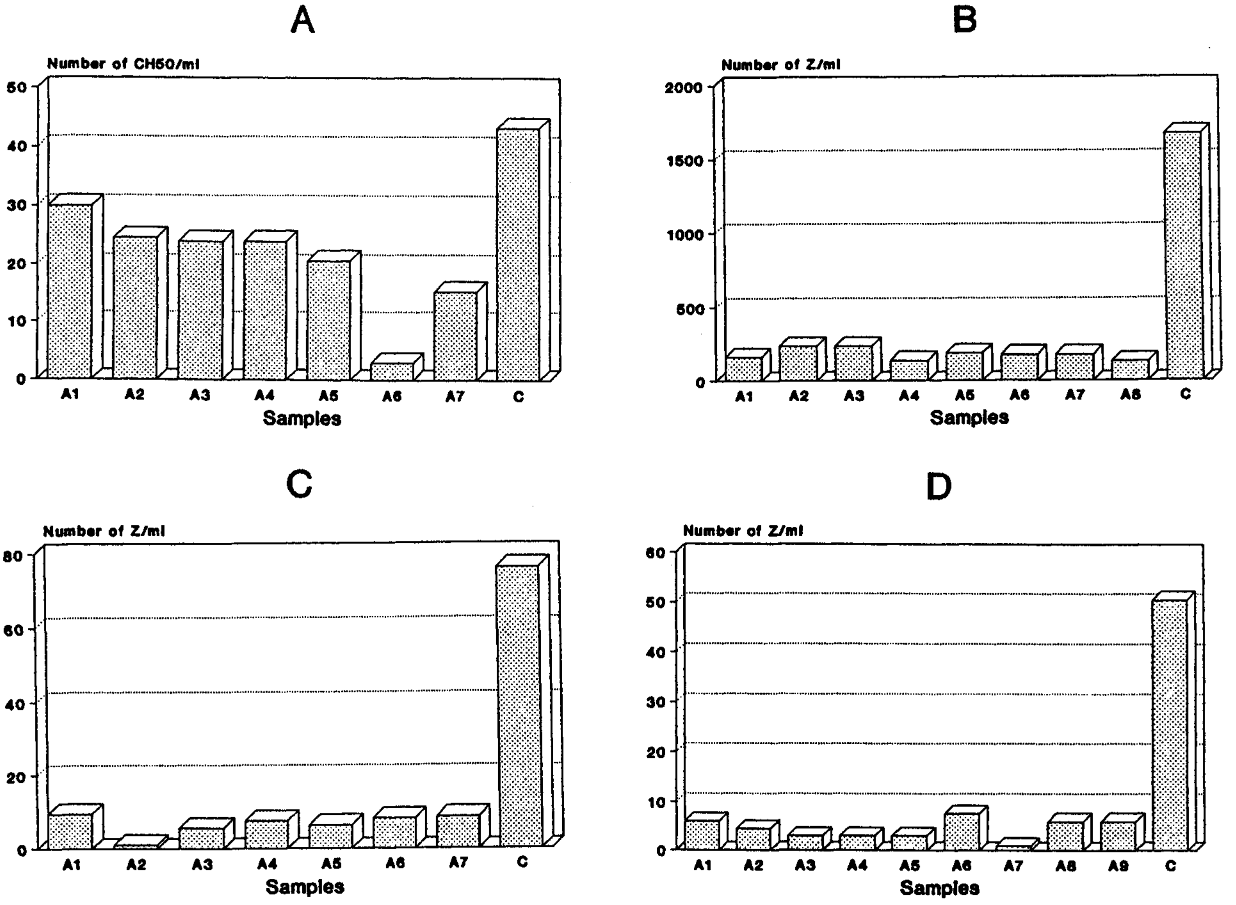

Fig. 1. Activation of human C system in NHS by trypomastigotes (TF) of *T. cruzi* pre-opsonized with the Abs A1 (IgM, mAb H3G6), A2 (IgM, mAb L1D10), A3 ($IgG_1$, mAb L2B11), A4 ($IgG_{2a}$ mAb 6A2), A5 ($IgG_1$, mAb B10.1), A6 (poly IgG anti-*T. cruzi*), A7 (total IMS anti-*T. cruzi*) or with NHS (control-C). Total C consumption was measured by determining the numbers of $CH_{50}$ remaining in the samples of NHS incubated with pre-opsonized TF using sheep red blood cells (E) presensitized with rabbit serum anti-E(EA) in pH 7.4 isotonic veronal buffered saline containing 0.1% gelatin, 15 mM $CaCl_2$ and 5 mM $MgCl_2$ ($VBS^{++}$) (panel A). The components C4 (panel B) and C3 (panel C) were measured by determining the number of haemolytically active molecules (Z) remaining in the samples of NHS incubated with pre-opsonized TF using EACI or EACI42 intermediate cells, respectively, in $VBS^{++}$ containing 2.5% glucose ($DGVBS^{++}$). Factor B (panel D) was measured by determining the number of haemolytically active molecules (Z) remaining in the samples of NHS incubated with pre-opsonized TF using rabbit blood cells ($E^r$) and NHS previously depleted of factor B in $DGVBS^{++}$ containing 1 mM Mg and 10 mM EGTA.

**Table 1.** C-dependent lysis of the trypomastigote forms of *T. cruzi* pre-opsonized with mouse monoclonal or polyclonal antibodies against *T. cruzi*

| Trypomastigotes of [a] *T. cruzi* optimized with: | % of killing |
|---|---|
| A1 (IgM mAb H3G6) | 6 |
| A2 (IgM mAb L1D10) | 5 |
| A3 (IgG1 mAb L2B11) | 2 |
| A4 (IgG2a mAb 6A2) | 7 |
| A5 (IgG1 mAb B10.1) | 8 |
| A6 (total IgG) | 50 |
| A7 (IMS) | 72 |
| A8 (IgM) | 65 |
| A9 (Fab$\mu$) | 39 |
| — | 0 |

[a] Trypomastigotes of *T. cruzi* were pre-opsonized with the indicated Abs for 30 min at 4°C and, after washing they were reincubated with NHS for 1 h at 37°C. The number of motile parasites were determined in a haemocytometer just before (time, O) and after the incubation (time, 1 h) and the percentage of reduction was calculated. Each number represents the mean of three experiments.

the classical pathway, also transforms trypomastigotes into ACP activators.[21]

Five mAbs that recognize and combine with epitopes present on the trypomastigote cell surface, although being able to activate the classical and the alternative C pathways, were devoid of lytic activity. In contrast, polyclonal anti-sera rich in lytic activity or their purified IgG, IgM fractions and the Fab$\mu$ fragments were able to sensitize trypomastigotes for total C, C4, B and C3 consumption, and lysis. This observed ability of the polyclonal Abs herein used cannot be due to its higher capacity to interact with specific epitopes, as compared with the monoclonal Abs, since recent reports have demonstrated that human monoclonal Abs with affinity values of $1.8 \times 10^8$ l/mol belonging to $IgG_1$, $IgG_3$ and IgM isotypes were able to activate the classical C pathway while those belonging to IgA, $IgG_2$ and to a lesser extent $IgG_4$, but never IgM, were able to activate the alternative pathway.[22]

Thus, all studied Abs were able to combine with epitopes belonging to the trypanosome cell surface but only some were endowed with the capacity to trigger C-mediating lysis of opsonized parasites. The C cascade activated by the Abs of the first group could be under restrict control of RPC-like molecules strategically distributed on the parasite cell surface or is unable to form, or to insert, the C5b–C9 complex into the parasite membrane. In contrast, the Abs of the second group either overpass the activity of the RPC-like molecules or form C5b–C9 complexes in conditions to be inserted in and hit the parasite membrane. Blockage of the RPC-like molecules, for instance the T-DAF, is the mechanism suggested for the trypanosome killing activity exhibited by the latter group of antibodies.

### References

1. Kipnis TL, Sher A, *et al.* Enzymatic treatment transforms trypomastigotes of *Trypanosoma cruzi* into activators of the alternative complement pathway and potentiates their uptake by macrophages. *Proc Natl Acad Sci USA* 1981; **78:** 602–605.
2. Kipnis TL, Tambourgi DV, *et al.* Effect of *Trypanosoma cruzi* membrane components on the formation of the classical pathway C3 convertase. *Brazilian J Med Biol Res* 1986; **19:** 271–278.
3. Stefani MMA, Takehara HA, Mota I. Isotypes antibodies responsible for immune lysis in *Trypanosoma cruzi* infected mice. *Immunol Lett* 1983; **7:** 91–97.
4. Rey-Campos J, Rubinstein P, Rodrigues de Cordoba S. A physical map of the human regulator of complement activation gene cluster linking the complement genes CR1, CR2, DAF and C4BP. *J Exp Med* 1988; **167:** 664–669.
5. Seya T, Ballard LL, *et al.* Distribution of membrane cofactor protein of complement on human peripheral blood cells. An altered form is found in granulocytes. *Eur J Immunol* 1988; **18:** 1289–1294.
6. Frank MM, Fries LF. The role of complement in defense against bacterial disease. *Baillière's Clinical Immunology and Allergy* 1988; **2:** 335–361.
7. Pangburn MK, Schreiber RD, Müller-Eberhard HJ. Human complement C3b inactivator: isolation, characterization, and demonstration of an absolute requirement for the serum protein BIH for cleavage of C3b and C4b in solution. *J Exp Med* 1977; **146:** 257–270.
8. Fujita T, Gigli I, Nussenzweig V. Human C4 binding protein II. Role in proteolysis of C4b by C3b inactivator. *J Exp Med* 1978; **148:** 1044–1051.
9. Fearon DT. Regulation of the amplification C3 convertase of human complement by an inhibitory protein isolated from human erythrocyte membrane. *Proc Natl Acad Sci USA* 1979; **76:** 5299–5302.
10. Nicholson-Weller A, Burge J, *et al.* Isolation of a human erythrocyte membrane glycoprotein with decay-accelerating activity for C3 convertases of the complement system. *J Immunol* 1982; **129:** 184–189.
11. Rimoldi MT, Sher A, *et al.* Developmentally regulated expression by *Trypanosoma cruzi* of molecules that accelerate the decay of complement C3 convertases. *Proc Natl Acad Sci USA* 1988; **85:** 193–197.
12. Joiner KA, Dias da Silva W, *et al.* Biochemical characterization of a factor produced by trypomastigotes of *Trypanosoma cruzi* that accelerates the decay of complement C3 convertases. *J Biol Chem* 1988; **263:** 11327–11335.
13. Tambourgi DV, Kipnis TL, *et al.* T-DAF: developmentally regulated complement inhibitor expressed by trypomastigotes of *T. cruzi*. *Mem Inst Oswaldo Cruz* 1991; **86:** 81–82.
14. Krettli AU, Brener Z. Resistance against *Trypanosoma cruzi* associated to anti-living trypomastigote antibodies. *J Immunol* 1982; **128:** 2209–2012.
15. Kipnis TL, Krettli AU. Transformation of trypomastigote bloodstream forms of *Trypanosoma cruzi* into activators of the alternative complement pathway by immune fragments. *Scand J Immunol* 1985; **22:** 217–226.
16. Alves MJM, Abuin G, *et al.* Partial inhibition of trypomastigote entry into cultured mammalian cells by monoclonal antibodies against a surface glycoprotein of *Trypanosoma cruzi*. *Mol Biochem Parasitol* 1986; **21:** 75–82.
17. Müller-Eberhard HJ, Hoffman LG, *et al.* Complement. In: Williams LA, Chase MW, eds. *Methods in Immunology and Immunochemistry*. 1977; 127–274.
18. Platts-Mills TAE, Ishizaka K. Activation of the alternative pathway of human complement by rabbit cells. *J Immunol* 1974; **113:** 348–354.
19. Lima-Martins MVC, Sanchez GA, *et al.* Antibody-dependent cell cytotoxicity against *Trypanosoma cruzi* is only mediated by protective antibodies. *Parasite Immunol* 1985; **7:** 367–372.
20. Lages-Silva E, Ramirez LE, *et al.* Effect of protective and non-protective antibodies in the phagocytosis rate of *Trypanosoma cruzi* blood forms by mouse peritoneal macrophages. *Parasite Immunol* 1987; **9:** 21–28.
21. Kipnis TL, Sucupira M, Dias da Silva W. Transformation of *Trypanosoma cruzi* trypomastigote bloodstream forms by immune IgM and its Fabμ fragment into activators of the alternative complement pathway. *Brazilian J Med Biol Res* 1987; **20:** 105–114.
22. Valim YLM, Lachmann PJ. The antibody isotypes pattern of complement activation depends on antigens epitopes density and antigen–antibody ratio *Clin Exp Immunol* 1991; **84:** 1–8.

ACKNOWLEDGEMENTS. We thank Elaine Rodrigues for preparing the manuscript. This work was supported by: Fundação de Amparo à Pesquisa do Estado de São Paulo 90-0162-7 and Conselho Nacional de Desenvolvimento Científico e Tecnológico 50-403/89-4.

**Received 12 June 1992; accepted in revised form 20 July 1992**
